# Case Report: JAK inhibitors for the treatment of anti-melanoma differentiation-associated gene 5-positive dermatomyositis with spontaneous pneumomediastinum

**DOI:** 10.3389/fmed.2025.1642484

**Published:** 2025-09-02

**Authors:** Xin Yang, Xiaoguang Cui, Xueyi Li

**Affiliations:** Department of Rheumatology, Xi’an Jiaotong University Second Affiliated Hospital, Xi’an, China

**Keywords:** anti-melanoma differentiation-associated gene 5 antibody, spontaneous pneumomediastinum, Janus kinase inhibitors, subcutaneous emphysema, interstitial lung disease

## Abstract

**Background:**

Spontaneous pneumomediastinum (SPNM) occurred more frequently in dermatomyositis (DM) patients with anti-melanoma differentiation-associated gene 5 (anti-MDA5) antibody than in DM patients without anti-MDA5 antibody. SPNM has been indicated as a risk factor for death in anti-MDA5-positive DM. There have been no clinical studies evaluating the treatment strategy for SPNM in anti-MDA5-positive DM.

**Methods:**

We present a series of five cases in which the administration of Janus kinase (JAK) inhibitors improved the clinical condition of anti-MDA5-positive DM patients with SPNM.

**Results:**

Clinical conditions of the four patients who were given JAK inhibitors after the diagnosis of SPNM were all improved. SPNM symptoms of the patient who was not commenced on JAK inhibitors progressed, and the patient died of severe pulmonary infection.

**Conclusion:**

JAK inhibitors appear to be an efficient therapeutic option in the treatment of anti-MDA5-positive DM with SPNM.

## Highlights

- JAK inhibitors should be considered in anti-MDA5-positive DM patients with SPNM.

## Introduction

Anti-melanoma differentiation-associated gene 5 (anti-MDA5) antibody-positive dermatomyositis (DM) is characterized by clinically amyopathic DM (CADM) and rapidly progressive interstitial lung disease (RP-ILD), which is correlated with an aggressive course and poor prognosis (1). Spontaneous pneumomediastinum (SPNM) is a condition characterized by free air around the mediastinal structures and is a relatively rare complication in patients with DM and ILD (2). The prevalence of SPNM in myositis was estimated to be 2.2–7.6% and tends to occur in DM patients with RP-ILD, anti-MDA5 antibody, CADM diagnosis, low creatine kinase (CK) level, and patients with cutaneous ulcers (2, 3). SPNM is a significant complication to be aware of, as there is evidence that it leads to higher mortality in patients with myositis; the mortality rate within the first month of diagnosis is as high as 25% (3, 4). The incidence of SPNM in anti-MDA5-positive DM patients reaches more than 15% and mortality is elevated up to approximately 60% (5). Combination therapy of high-dose glucocorticoids and cyclophosphamide, tacrolimus, JAK inhibitors, or rituximab is used in anti-MDA5-positive DM patients with ILD (6). However, there have been no treatment guidelines for SPNM in anti-MDA5-positive DM patients. In the following case series, we describe the therapeutic effect of JAK inhibitors on anti-MDA5-positive DM complicated by SPNM. Characteristics of the five patients are shown in Table 1.

**Table 1:** Characteristics of patients with anti-MDA5-positive DM complicated by interstitial lung disease and SPNM.

Patient	Patient 1	Patient 2	Patient 3	Patient 4	Patient 5
Sex, male/female	female	male	male	male	female
Age at the onset of DM, years	50	67	29	53	32
Heliotrope rash	−	−	−	+	+
Gottron’s sign rash	+	−	+	+	+
Skin ulcer	−	−	−	−	−
Shawl-sign rash	−	+	−	+	+
V-sign rash	+	+	−	+	+
Muscle weakness	−	−	−	+	+
Subcutaneous emphysema	+	−	+	+	−
Interval between DM onset and the occurrence of SPNM, months	8	27	4	13	14
ILD	+	+	+	+	+
RP-ILD	+	−	+	−	−
Treatment before the onset of SPNM					
Cyclophosphamide	+	+	−	+	−
Mycophenolate	−	−	+	−	−
Tacrolimus	+	+	+	−	+
Ciclosporin	−	−	−	−	−
Tofacitinib	−	+	+	−	−
Baricitinib	+	−	−	−	+
Pirfenidone	+	+	+	−	−
Duration of FUP, months	33	15	52	2	13
Outcome	Alive	Alive	Alive	Dead	Alive
Laboratory tests (at diagnosis of SPMN)					
ANA	−	−		−	−
RO52	+	−		+	−
CK, IU/L	24	33	33	114	32
LDH, IU/L	338	213	214	496	436
Ferritin, ng/mL	118	NA	644	3,815	306
PaO2, mmHg	56	NA	93	87	NA
ESR, mm/h	17	9	19	128	47.2
CRP, mg/L	11.3	2	2.6	41.4	2.4

Anti-MDA5-positive DM: anti-melanoma differentiation-associated gene 5 antibody-positive dermatomyositis; ILD, interstitial lung disease, RP-ILD, rapidly progressive interstitial lung disease; SPNM, spontaneous pneumomediastinum; FUP, follow-up; ANA, antinuclear antibody; LDH, lactate dehydrogenase; ESR, erythrocyte sedimentation rate; CRP, C-reactive protein.

## Methods

We carried out a retrospective survey to identify cases of anti-MDA5-positive DM complicated with SPNM hospitalized in the Department of Rheumatology at the Second Affiliated Hospital of Xi’an Jiaotong University from October 2018 to December 2023. Cases of traumatic and iatrogenic pneumomediastinum were excluded. Survival status was confirmed by hospital records or the follow-up calls. All patients were screened for a panel of myositis-specific antibodies and myositis-associated antibodies (anti-OJ, EJ, PL7, PL12, SRP, JO-1, MDA5, TIF1-*γ*, Mi-2α, Mi-2β, Ku, NXP2, pneumomediastinum (PM)-Scl75, PM-Scl100, SAE1, and Ro52 antibodies) using a commercial immunoblot assay. A diagnosis of anti-MDA5-positive DM was based on the Bohan and Peter criteria (7, 8) or the 239th European Neuromuscular Centre (ENMC) criteria (9). RP-ILD was characterized by progressive dyspnea and hypoxemia, with a worsening of radiologic changes of interstitial lung inflammation within 3 months after the onset of respiratory symptoms (10). Among 61 anti-MDA5-positive DM patients, 5 patients were complicated with SPNM.

## Results

### Patient 1

**Figure 1:**
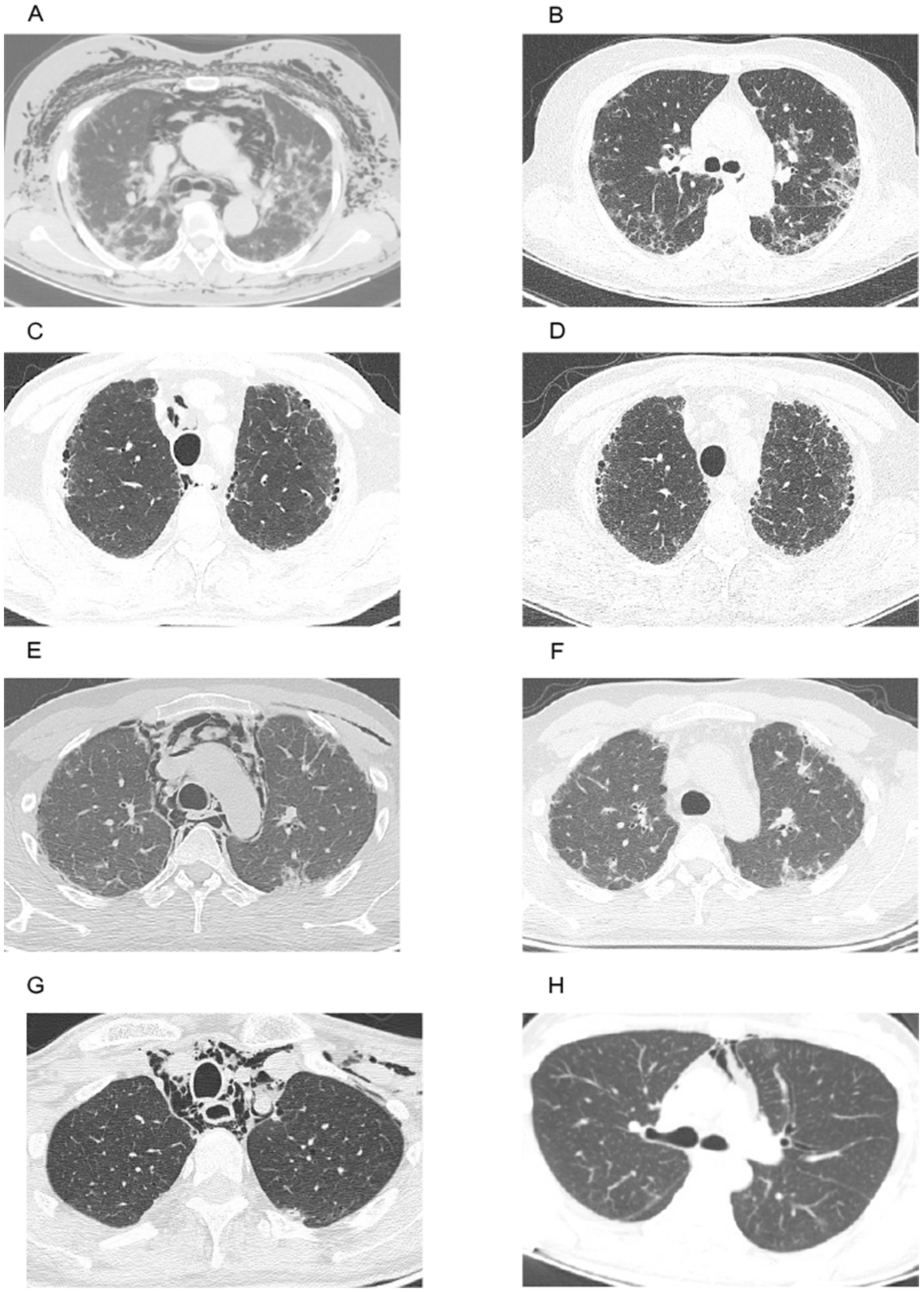
**(A)** Chest CT scan revealed bibasilar consolidations indicating interstitial lung disease, extensive pneumomediastinum, and subcutaneous emphysema. **(B)** Chest CT scan after combination therapy with tofacitinib, pneumomediastinum, and subcutaneous emphysema resolved. **(C)** Chest CT scan revealed ground-glass opacities and pneumomediastinum. **(D)** Chest CT scan after combination therapy with tofacitinib, pneumomediastinum resolved. **(E)** Chest CT scan revealed multiple bilateral peripheral and subpleural areas of consolidation. **(F)** Chest CT scan after combination therapy with tofacitinib, pneumomediastinum, and subcutaneous emphysema resolved. **(G)** Chest CT scan revealed bibasilar consolidations and extensive pneumomediastinum and subcutaneous emphysema. **(H)** Chest CT scan revealed pneumomediastinum.

A 50-year-old woman presented with a skin rash, progressive dyspnea, and cough for 2 months. Physical examination showed facial rash and Gottron papules over the metacarpal and interphalangeal joints. The patient did not have myalgias or muscle weakness. Her chemistry panel showed an elevated erythrocyte sedimentation rate (ESR) [42 mm/h (normal: 0–20 mm/h)] and the lactate dehydrogenase (LDH) level [301 IU/L (normal: 120–250 IU/L)]. The patient tested positive for anti-Ro52 IgG (+++). The remaining routine panel was unremarkable. Screening for myositis-specific antibodies and myositis-associated antibodies revealed strong positivity for the anti-MDA5 antibody. Chest CT revealed signs of ILD. Given these findings, the diagnosis of anti-MDA5-positive DM was confirmed. She was initially treated with prednisone (1 mg/kg/day), intravenous cyclophosphamide (600 mg, every 2 weeks), oral tacrolimus (1 mg/day), and pirfenidone (400 mg, three times daily). Her symptoms (i.e., dyspnea and cough) improved after 2 weeks. Cyclophosphamide was discontinued after 3 months. However, 6 months after initial treatment, as prednisone was tapered down to 10 mg daily, she was admitted to the emergency room (ER) due to progressive dyspnea, worsening subcutaneous emphysema, and fever for 1 week. Physical examination revealed a crepitus sensation upon palpation of the neck and chest skin. The chest CT revealed markedly increased bibasilar consolidations indicating progression of interstitial lung disease, extensive pneumomediastinum, and subcutaneous emphysema (Figure 1A). C-reactive protein (CRP 11.3 mg/L) was also significantly elevated. The diagnosis of SPNM was made. After 1 week of empirical anti-infective therapy, baricitinib (2 mg/day) was added to the treatment regimen, and intravenous cyclophosphamide was reintroduced (600 mg, every 2 weeks for six doses). Prednisone, tacrolimus, and pirfenidone were continued. Her shortness of breath and rash improved afterward. A high-resolution computed tomography (HRCT) performed 2 years later showed complete resolution of the pneumomediastinum (Figure 1B). Baricitinib was maintained until the last follow-up.

### Patient 2

A 67-year-old man was admitted to our hospital with a 2-year history of worsening dyspnea and cough. Physical examination on admission showed Shawl-sign rash and V-sign rash. Routine blood work revealed increased levels of ESR (43 mm/h [normal range <20 mm/h]). No other significant abnormalities were noted. Myositis-specific antibodies and myositis-associated antibody tests were positive for anti-MDA5 antibody. Chest CT revealed signs of ILD. Consequently, he was diagnosed with anti-MDA5-positive DM. He was commenced on prednisone (1 mg/kg/day), cyclophosphamide (600 mg, every 2 weeks for six doses), tacrolimus (1 mg/day), and pirfenidone (400 mg, three times daily). Under this therapeutic regimen, his dyspnea abated. Chest CT was ordered 3 months after initial treatment, showing pneumomediastinum (Figure 1C). However, worsening symptoms such as cough or shortness of breath were not reported. ESR, CRP, and CK were all within normal limits. No additional evidence of disease progression was identified apart from pneumomediastinum. Tofacitinib (5 mg, twice daily) was started in combination with prednisone, tacrolimus (1 mg twice daily), and pirfenidone (400 mg three times daily). Six months later, the patient’s HRCT findings improved significantly (Figure 1D). At the last follow-up, the patient remained on the treatment of prednisone, tacrolimus, tofacitinib, and pirfenidone.

### Patient 3

A 30-year-old man presented with a 1-month history of cough, fever, and skin rash. Physical examination revealed Gottron papules over the interphalangeal and elbow joints. Routine blood work revealed elevated levels of serum alanine aminotransferase (ALT) (96 IU/L [normal range 9–50 IU/L]), aspartate aminotrasferase (AST)(102 IU/L [normal range 15–40 IU/L]), serum gamma-glutamyl transferase (GGT) (89 IU/L [normal range 10–60 IU/L]), and ferritin (1,541 ng/mL [normal: 13–150 ng/mL]). Arterial blood gases at rest showed decreased PaO2 (68.2 mmHg) and normal PaCO2 (39.6 mmHg). Anti-MDA5 antibody was detected in the myositis-specific antibodies and myositis-associated antibody panels. Chest CT revealed signs of ILD. A diagnosis of anti-MDA5-positive DM was made. He was given steroid pulse therapy (methylprednisolone 1,000 mg, consecutively for 3 days) followed by oral prednisone (1 mg/kg/day) and mycophenolate (750 mg twice daily). The patient’s symptoms improved gradually. Three months after initial treatment, he visited the ER with complaints of chest pain and subcutaneous emphysema with sudden-onset shortness of breath. Room air arterial blood gases revealed normal PaO2 and normal PaCO2. The blood work revealed increased serum GGT (180 IU/L [normal range: 10–60 IU/L]) and ferritin (644 ng/mL [normal: 13–150 ng/mL]). The chest CT demonstrated pneumomediastinum and subcutaneous emphysema, and multiple newly developed consolidations in the subpleural region of the right upper lobe were also noted (Figure 1E). Based on the diagnosis of SPNM, we started treatment with methylprednisolone, tacrolimus (1 mg/day), and tofacitinib (5 mg, twice daily). Pirfenidone (400 mg, three times daily) was initiated after 1 month. Three months later, pneumomediastinum and subcutaneous emphysema resolved completely, as shown in Figure 1F. With the disease in remission, the patient discontinued all medications 24 months later.

### Patient 4

A 53-year-old man presented with progressively proximal muscle weakness, skin rash, dyspnea, and oral ulcers. One year ago, he was diagnosed with anti-MDA5-positive DM complicated by ILD after presenting with skin rash, proximal muscle weakness, dyspnea, and dysphagia. He was initially treated with methylprednisolone (0.8 mg/kg/day) alone. After initial treatment with steroids, all his symptoms improved. However, his condition deteriorated when methylprednisolone was tapered to 20 mg daily. The patient was admitted to our hospital due to shortness of breath and cough with yellow sputum. Worsening of muscle weakness, skin rash, and oral ulcers were also reported. Physical examination revealed erythemas on the upper eyelids, neck, and shoulders. Crepitus on the neck was detected upon palpation. The chemistry panel showed elevated AST [10,264.5 IU/L (normal range 15–40 IU/L)], LDH [496 IU/L (normal: 120–250 IU/L)], GGT [583 IU/L (normal range 10–60 IU/L)], and ALP [242 IU/L (normal: 45–125 IU/L)]. Increased levels of ESR [128 mm/h (normal: 0–20 mm/h)], CRP (41.4 mg/L), and ferritin [3,815 ng/mL (normal: 13–150 ng/mL)] were observed. Room air arterial blood gases revealed normal PaO2 and normal PaCO2. Chest CT confirmed the finding of extensive pneumomediastinum, subcutaneous emphysema, and ILD (Figure 1G). Given the diagnosis of SPNM, we started therapy with methylprednisolone (1 mg/day) and oral cyclophosphamide (50 mg/day) combined with empirical anti-infective therapy. The patient declined the use of JAK inhibitors and other immunosuppressive agents due to a lack of health insurance coverage. There was no obvious improvement in his symptoms after treatment. Two months later, the patient died due to pulmonary infection and respiratory failure.

### Patient 5

A 32-year-old woman was admitted to our hospital with complaints of pruritic facial rash, myalgia, and muscle weakness. Physical examination showed facial rash, heliotrope rash, V-sign rash, Shawl-sign rash, and Gottron papules/signs over the metacarpal and interphalangeal joints. Her chemistry panel showed elevated ESR [47.2 mm/h (normal: 0–20 mm/h)], LDH [436 IU/L (normal: 120–250 IU/L)], and ferritin [306 ng/mL (normal: 13–150 ng/mL)]. The initial chest CT showed pneumomediastinum and ILD (Figure 1H). Serology for myositis-specific antibodies was positive for anti-MDA5 antibody. A diagnosis of anti-MDA5-positive DM was made. Prednisone (1 mg/kg/day), tacrolimus (1 mg/day), and baricitinib (2 mg/day) were initiated. She reported significant clinical improvement upon telephone follow-up 6 months after discharge. During the most recent telephone follow-up, the patient reported stable disease status and complete resolution of pneumomediastinum on HCRT performed 1 year after initial treatment. Baricitinib and tacrolimus were maintained until the last follow-up.

## Discussion

The occurrence of SPNM was developed by the rupture of the alveoli due to either raised intra-alveolar pressure in the presence of ILD and pulmonary vasculitis or weakened alveolar walls by corticosteroids (4). SPNM has been more commonly described among DM/PM patients than among patients with other connective tissue diseases. In a previous report, the prevalence of SPNM in patients with PM/ DM and in those with DM was estimated to be 7.6 and 11.8%, respectively, which is higher than that in the report by Le Goff et al. (3). SPNM occurred more frequently in anti-MDA5 antibody-positive than in anti-MDA5 antibody-negative myositis patients. A total of 10 out of 11 patients with dermatomyositis complicated by interstitial lung disease and SPNM were anti-MDA5 positive (2). The incidence of SPNM in this subtype of CADM was approximately 15.04% (5).

SPNM has been considered a poor prognostic factor in anti-MDA5-positive DM patients complicated with ILD (4). In the recent study, the mortality rate was significantly higher in anti-MDA5-positive DM patients with SPNM occurrence (53.3%) than in those without SPNM occurrence (4.0%), indicating that SPNM occurrence may warrant more intense treatment therapy (11).

At present, there are no consensus recommendations for the management of SPNM in MDA5 + DM patients. Several cases have been reported that SPNM patients with refractory ILD got remission after the use of combined immunosuppressive therapy (12, 13). Another successful case found that a lung transplant might be useful for PNM in MDA5 + DM patients (14). Additionally, an observational study by De Giacomi et al. (15) reported that no deaths of PNM in CTDs were observed after receiving the measures of observation only, oxygen therapy, and chest tube drainage.

Tofacitinib has been used as a promising immunosuppressant in treating patients with anti-MDA5-positive DM. In a single-center, open-label clinical study, anti-MDA5-positive DM-ILD patients treated with tofacitinib exhibited significantly higher overall survival (OS) compared with historical controls (16). Additionally, Kurasawa et al.’s (17) research revealed a better survival rate of the refractory anti-MDA5-positive DM-ILD patients who were treated with the combination therapy, including tofacitinib, than that of the historical controls. The efficacy of baricitinib was supported by a recent study (18).

Recently, multidisciplinary therapy, including tofacitinib, was reported to successfully treat an anti-MDA5-positive DM patient with SPNM (19). In our cases, 4 out of 5 SPNM patients were treated with combination therapy including JAK inhibitors, and all of these patients’ clinical conditions improved. One patient who was not commenced on JAK inhibitors died 2 months after the occurrence of SPNM. Our results indicated that JAK inhibitors may be promising drugs for anti-MDA5-positive DM patients with SPNM.

## Data Availability

The original contributions presented in the study are included in the article/supplementary material, further inquiries can be directed to the corresponding author.
